# Crystal structure of bis­(4-acetyl­anilinium) tetra­chlorido­cobaltate(II)

**DOI:** 10.1107/S2056989015021404

**Published:** 2015-11-18

**Authors:** Manickam Thairiyaraja, Arumugam Elangovan, Ramasamy Shanmugam, Kuthambalam Selvaraju, Subbiah Thamotharan

**Affiliations:** a PG & Research Department of Physics, Government Arts College, Ariyalur 621 713, India; b Department of Chemistry, Thiagarajar College, Madurai 625 009, India; c Biomolecular Crystallography Laboratory, Department of Bioinformatics, School of Chemical and Biotechnology, SASTRA University, Thanjavur 613 401, India

**Keywords:** crystal structure, isotypism, cobalt(II), hydrogen bonding, π–π stacking inter­actions

## Abstract

The structure of the title salt, (C_8_H_10_NO)_2_[CoCl_4_], is isotypic with the analogous cuprate(II) structure. The asymmetric unit contains one 4-acetyl­anilinium cation and one half of a tetra­chlorido­cobaltate(II) anion for which the Co^II^ atom and two Cl^−^ ligands lie on a mirror plane. The Co—Cl distances in the distorted tetra­hedral anion range from 2.2519 (6) to 2.2954 (9) Å and the Cl—Co—Cl angles range from 106.53 (2) to 110.81 (4)°. In the crystal, cations are self-assembled by inter­molecular N—H⋯O hydrogen-bonding inter­actions, leading to a *C*(8) chain motif with the chains running parallel to the *b* axis. π–π stacking inter­actions between benzene rings, with a centroid-to-centroid distance of 3.709 Å, are also observed along this direction. The CoCl_4_^2−^ anions are sandwiched between the cationic chains and inter­act with each other through inter­molecular N—H⋯Cl hydrogen-bonding inter­actions, forming a three-dimensional network structure.

## Related literature   

For the structure of the isotypic tetra­chlorido­cuprate(II) compound, see: Elangovan *et al.* (2007 ▸).
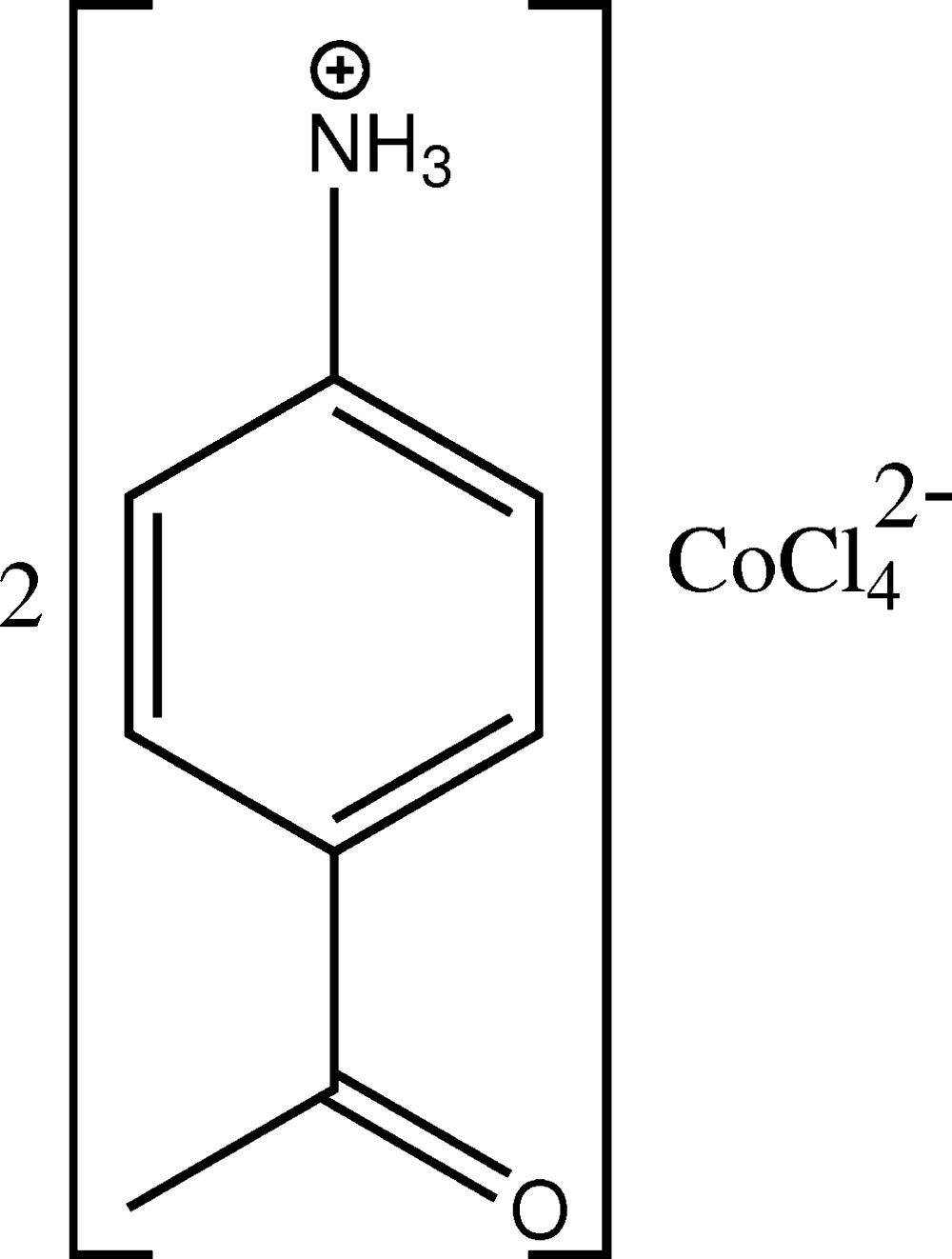


## Experimental   

### Crystal data  

(C_8_H_10_NO)_2_[CoCl_4_]
*M* *_r_* = 473.07
Orthorhombic, 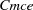

*a* = 19.4605 (6) Å
*b* = 15.5108 (6) Å
*c* = 13.7374 (5) Å
*V* = 4146.6 (3) Å^3^
*Z* = 8
Mo *K*α radiation
μ = 1.36 mm^−1^
*T* = 293 K
0.3 × 0.2 × 0.2 mm

### Data collection  

Bruker SMART APEX CCD diffractometer
Absorption correction: multi-scan (*SADABS*; Bruker, 2004 ▸) *T*_min_ = 0.687, *T*_max_ = 0.773
24499 measured reflections
3329 independent reflections
2439 reflections with *I* > 2σ(*I*)
*R*_int_ = 0.035

### Refinement  

*R*[*F* ^2^ > 2σ(*F* ^2^)] = 0.039
*wR*(*F* ^2^) = 0.123
*S* = 1.05
3329 reflections
131 parameters
3 restraints
H atoms treated by a mixture of independent and constrained refinement
Δρ_max_ = 1.35 e Å^−3^
Δρ_min_ = −0.66 e Å^−3^

### 

Data collection: *APEX2* (Bruker, 2004 ▸); cell refinement: *SAINT* (Bruker, 2004 ▸); data reduction: *SAINT*; method used to solve structure: coordinates taken from an isotypic structure; program(s) used to refine structure: *SHELXL2014* (Sheldrick, 2015 ▸); molecular graphics: *PLATON* (Spek, 2009 ▸); software used to prepare material for publication: *publCIF* (Westrip, 2010 ▸).

## Supplementary Material

Crystal structure: contains datablock(s) I, New_Global_Publ_Block. DOI: 10.1107/S2056989015021404/wm5237sup1.cif

**Fig. 1.**
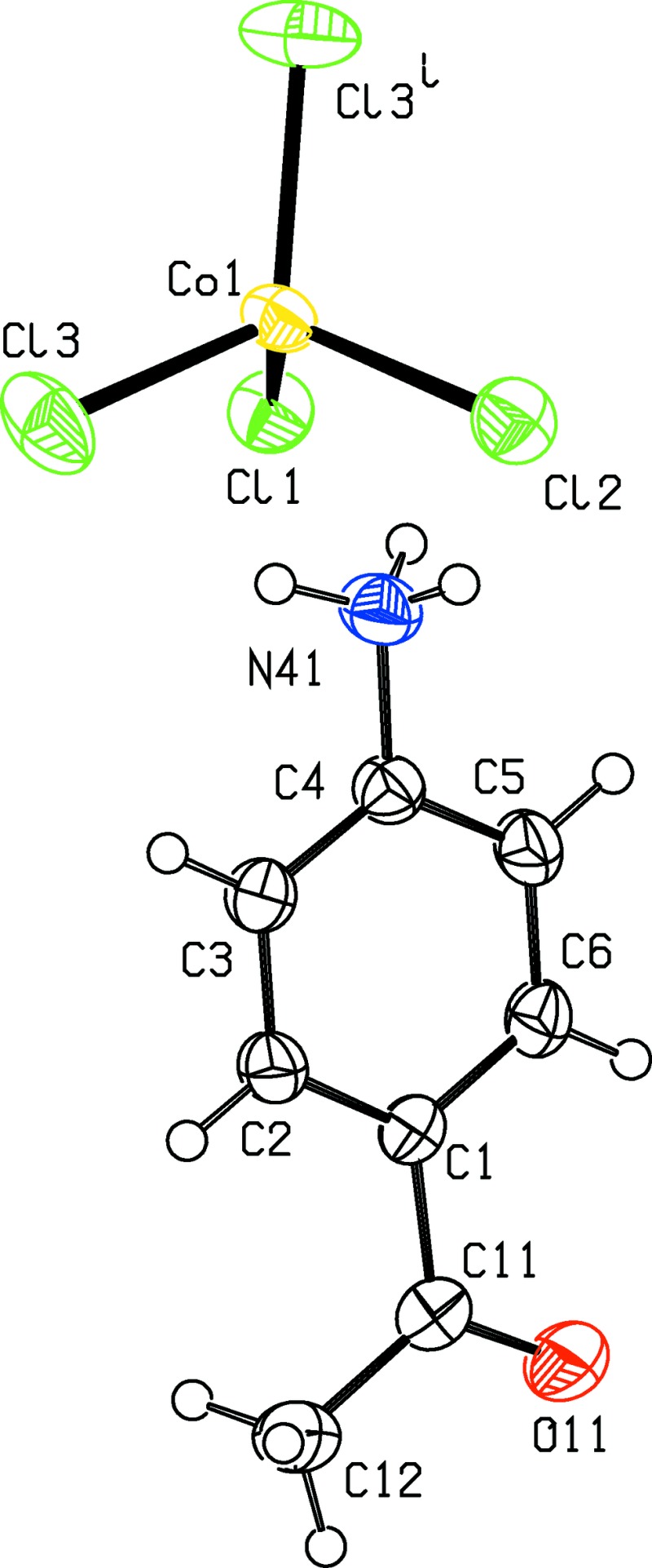
The molecular components in the structure of the title salt. Displacement ellipsoids are drawn at the 50% probability level. [Symmetry code: (i) -x, y, z.]

**Fig. 2.**
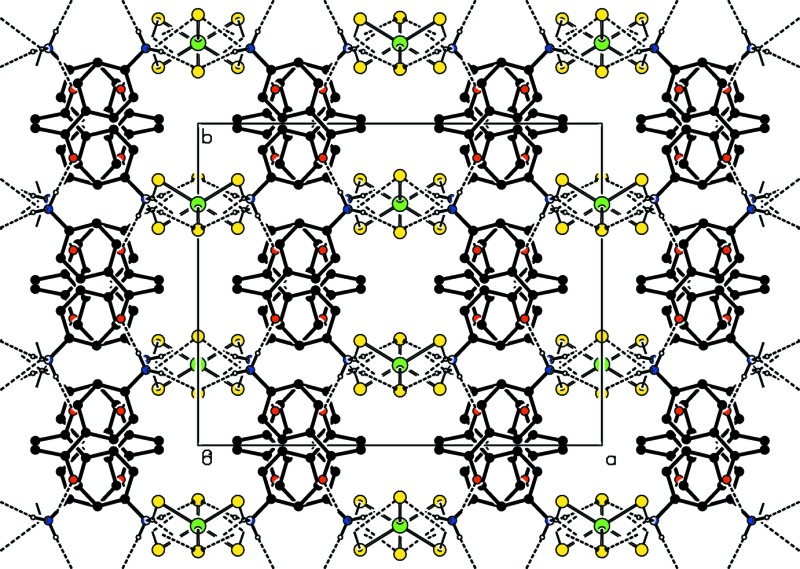
The crystal packing of the title salt viewed along the c axis. Hydrogen bonds are shown as dashed lines; H atoms bound to C were omitted for clarity.

Structure factors: contains datablock(s) I. DOI: 10.1107/S2056989015021404/wm5237Isup2.hkl

x y z . DOI: 10.1107/S2056989015021404/wm5237fig1.tif
The mol­ecular components in the structure of the title salt. Displacement ellipsoids are drawn at the 50% probability level. [Symmetry code: (i) −*x*, *y*, *z*.]

c . DOI: 10.1107/S2056989015021404/wm5237fig2.tif
The crystal packing of the title salt viewed along the *c* axis. Hydrogen bonds are shown as dashed lines; H atoms bound to C were omitted for clarity.

CCDC reference: 967676

Additional supporting information:  crystallographic information; 3D view; checkCIF report

## Figures and Tables

**Table 1:** Hydrogen-bond geometry (Å, °)

*D*—H⋯*A*	*D*—H	H⋯*A*	*D*⋯*A*	*D*—H⋯*A*
N41—H41*A*⋯O11^i^	0.91 (2)	1.88 (2)	2.781 (3)	174 (3)
N41—H41*B*⋯Cl2^ii^	0.92 (2)	2.31 (2)	3.211 (2)	168 (3)
N41—H41*C*⋯Cl3^iii^	0.88 (2)	2.48 (2)	3.309 (3)	157 (3)

Symmetry codes: (i) 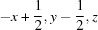; (ii) 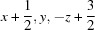; (iii) 
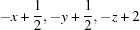
.

## supplementary crystallographic information

### S1. Synthesis and crystallization

A solution of 4-amino­aceto­phenone (20 mmol) in 2 ml of HCl and deionized water (10 ml) was added to a 10 ml solution of CoCl_2_·6H_2_O (10 mmol). The resulting solution was concentrated and kept unperturbed at ambient temperature for crystallization. Dark green block-shaped crystals were obtained after 7 days.

### S2. Refinement

Since the title complex is isotypic with its tetra­chloridocuprate counterpart, it was refined with the coordinates of the latter (Elangovan *et al.*, 2007) as starting parameters. The amino H atoms were located from a difference Fourier map and refined with a distance restraint of N—H = 0.89 (2) Å. The methyl H atoms were constrained to an ideal geometry (C—H = 0.96 Å) with *U*_iso_(H) = 1.5*U*_eq_(C), but were allowed to rotate freely about the C–C bond. The remaining H atoms were positioned in geometrically calculated positions and refined using a riding model with C—H = 0.93 Å and *U*_iso_(H) = 1.2*U*_eq_(C). At this stage, the maximum residual electron density of 1.35 e Å^-3^ indicated the presence of a possible atom at Wyckofff position 4*a* at a distance of 2.81Å near atom H5. This peak was assumed to be the O atom of a water molecule and was refined with isotropic displacement parameters. However, the resultant model had slightly higher reliability factors and a very high isotropic atomic displacement parameter for this O atom. As a consequence, this water O atom was not included in the final model.

### Crystal data

**Table:**

(C_8_H_10_NO)_2_[CoCl_4_]	*D*_x_ = 1.516 Mg m^−^^3^
*M**_r_* = 473.07	Mo *K*α radiation, λ = 0.71073 Å
Orthorhombic, *C**m**c**e*	Cell parameters from 14227 reflections
*a* = 19.4605 (6) Å	θ = 2.0–30.0°
*b* = 15.5108 (6) Å	µ = 1.36 mm^−^^1^
*c* = 13.7374 (5) Å	*T* = 293 K
*V* = 4146.6 (3) Å^3^	Block, green
*Z* = 8	0.3 × 0.2 × 0.2 mm
*F*(000) = 1928	

### Data collection

**Table:**

Bruker SMART APEX CCD diffractometer	2439 reflections with *I* > 2σ(*I*)
Radiation source: fine-focus sealed tube	*R*_int_ = 0.035
ω and φ scan	θ_max_ = 30.9°, θ_min_ = 2.2°
Absorption correction: multi-scan (*SADABS*; Bruker, 2004)	*h* = −27→28
*T*_min_ = 0.687, *T*_max_ = 0.773	*k* = −18→22
24499 measured reflections	*l* = −19→18
3329 independent reflections	

### Refinement

**Table:**

Refinement on *F*^2^	Primary atom site location: isomorphous structure methods
Least-squares matrix: full	Hydrogen site location: mixed
*R*[*F*^2^ > 2σ(*F*^2^)] = 0.039	H atoms treated by a mixture of independent and constrained refinement
*wR*(*F*^2^) = 0.123	*w* = 1/[σ^2^(*F*_o_^2^) + (0.0558*P*)^2^ + 6.0921*P*] where *P* = (*F*_o_^2^ + 2*F*_c_^2^)/3
*S* = 1.05	(Δ/σ)_max_ < 0.001
3329 reflections	Δρ_max_ = 1.35 e Å^−^^3^
131 parameters	Δρ_min_ = −0.66 e Å^−^^3^
3 restraints	

### Special details

**Table:**

Geometry. All e.s.d.'s (except the e.s.d. in the dihedral angle between two l.s. planes) are estimated using the full covariance matrix. The cell e.s.d.'s are taken into account individually in the estimation of e.s.d.'s in distances, angles and torsion angles; correlations between e.s.d.'s in cell parameters are only used when they are defined by crystal symmetry. An approximate (isotropic) treatment of cell e.s.d.'s is used for estimating e.s.d.'s involving l.s. planes.

### Fractional atomic coordinates and isotropic or equivalent isotropic displacement parameters (Å^2^)

**Table:**

	*x*	*y*	*z*	*U*_iso_*/*U*_eq_	
O11	0.19113 (10)	0.60656 (11)	0.87507 (15)	0.0548 (5)	
N41	0.37078 (11)	0.26577 (13)	0.90637 (18)	0.0421 (5)	
H41A	0.3519 (18)	0.2142 (15)	0.891 (3)	0.082 (12)*	
H41B	0.4081 (14)	0.276 (2)	0.867 (2)	0.068 (10)*	
H41C	0.3860 (16)	0.268 (2)	0.9661 (14)	0.065 (10)*	
C1	0.22194 (11)	0.46100 (12)	0.87145 (14)	0.0299 (4)	
C2	0.20229 (11)	0.37474 (14)	0.86968 (16)	0.0353 (4)	
H2	0.1563	0.3603	0.8613	0.042*	
C3	0.25125 (12)	0.30994 (13)	0.88036 (16)	0.0366 (5)	
H3	0.2384	0.2522	0.8793	0.044*	
C4	0.31896 (11)	0.33283 (13)	0.89257 (15)	0.0322 (4)	
C5	0.33997 (11)	0.41822 (14)	0.89347 (17)	0.0371 (5)	
H5	0.3861	0.4324	0.9011	0.045*	
C6	0.29092 (11)	0.48178 (13)	0.88278 (16)	0.0358 (4)	
H6	0.3042	0.5394	0.8832	0.043*	
C11	0.17101 (12)	0.53274 (14)	0.86411 (15)	0.0352 (4)	
C12	0.09695 (12)	0.51393 (16)	0.8452 (2)	0.0483 (6)	
H12A	0.0727	0.5670	0.8343	0.072*	
H12B	0.0776	0.4848	0.9004	0.072*	
H12C	0.0929	0.4779	0.7886	0.072*	
Co1	0.0000	0.25104 (3)	0.86031 (3)	0.03357 (13)	
Cl1	0.0000	0.33842 (5)	0.99225 (6)	0.04207 (19)	
Cl2	0.0000	0.33034 (6)	0.71924 (6)	0.0462 (2)	
Cl3	0.09841 (3)	0.17486 (5)	0.86639 (6)	0.0601 (2)	

### Atomic displacement parameters (Å^2^)

**Table:**

	*U*^11^	*U*^22^	*U*^33^	*U*^12^	*U*^13^	*U*^23^
O11	0.0497 (10)	0.0283 (8)	0.0863 (14)	0.0022 (7)	−0.0056 (9)	−0.0036 (8)
N41	0.0326 (10)	0.0337 (10)	0.0599 (14)	0.0014 (8)	−0.0043 (9)	0.0067 (9)
C1	0.0330 (9)	0.0256 (8)	0.0311 (10)	−0.0005 (7)	0.0005 (8)	−0.0006 (7)
C2	0.0280 (9)	0.0304 (9)	0.0476 (12)	−0.0044 (8)	−0.0006 (8)	0.0006 (8)
C3	0.0337 (10)	0.0261 (9)	0.0499 (12)	−0.0046 (8)	−0.0011 (9)	0.0022 (8)
C4	0.0311 (10)	0.0297 (9)	0.0358 (10)	0.0002 (8)	−0.0012 (8)	0.0030 (8)
C5	0.0306 (10)	0.0329 (10)	0.0479 (12)	−0.0054 (8)	−0.0054 (9)	0.0005 (9)
C6	0.0371 (11)	0.0270 (9)	0.0432 (12)	−0.0060 (8)	−0.0026 (9)	−0.0009 (8)
C11	0.0387 (11)	0.0310 (9)	0.0360 (11)	0.0017 (8)	0.0020 (9)	−0.0008 (8)
C12	0.0355 (12)	0.0417 (12)	0.0676 (17)	0.0053 (10)	0.0028 (11)	−0.0038 (11)
Co1	0.0253 (2)	0.0355 (2)	0.0399 (2)	0.000	0.000	−0.00463 (16)
Cl1	0.0469 (4)	0.0377 (4)	0.0416 (4)	0.000	0.000	−0.0080 (3)
Cl2	0.0456 (4)	0.0525 (5)	0.0405 (4)	0.000	0.000	0.0007 (3)
Cl3	0.0399 (3)	0.0581 (4)	0.0824 (5)	0.0193 (3)	−0.0109 (3)	−0.0218 (3)

### Geometric parameters (Å, º)

**Table:**

O11—C11	1.220 (3)	C4—C5	1.386 (3)
N41—C4	1.461 (3)	C5—C6	1.380 (3)
N41—H41A	0.904 (18)	C5—H5	0.9300
N41—H41B	0.921 (18)	C6—H6	0.9300
N41—H41C	0.872 (17)	C11—C12	1.493 (3)
C1—C6	1.389 (3)	C12—H12A	0.9600
C1—C2	1.392 (3)	C12—H12B	0.9600
C1—C11	1.494 (3)	C12—H12C	0.9600
C2—C3	1.393 (3)	Co1—Cl3^i^	2.2519 (6)
C2—H2	0.9300	Co1—Cl3	2.2519 (6)
C3—C4	1.375 (3)	Co1—Cl1	2.2631 (9)
C3—H3	0.9300	Co1—Cl2	2.2954 (9)
			
C4—N41—H41A	109 (2)	C4—C5—H5	120.7
C4—N41—H41B	110 (2)	C5—C6—C1	120.96 (19)
H41A—N41—H41B	110 (3)	C5—C6—H6	119.5
C4—N41—H41C	109 (2)	C1—C6—H6	119.5
H41A—N41—H41C	112 (3)	O11—C11—C12	121.0 (2)
H41B—N41—H41C	106 (3)	O11—C11—C1	118.6 (2)
C6—C1—C2	119.37 (19)	C12—C11—C1	120.44 (19)
C6—C1—C11	118.42 (18)	C11—C12—H12A	109.5
C2—C1—C11	122.2 (2)	C11—C12—H12B	109.5
C1—C2—C3	120.3 (2)	H12A—C12—H12B	109.5
C1—C2—H2	119.9	C11—C12—H12C	109.5
C3—C2—H2	119.9	H12A—C12—H12C	109.5
C4—C3—C2	118.82 (19)	H12B—C12—H12C	109.5
C4—C3—H3	120.6	Cl3^i^—Co1—Cl3	116.52 (4)
C2—C3—H3	120.6	Cl3^i^—Co1—Cl1	106.53 (2)
C3—C4—C5	122.04 (19)	Cl3—Co1—Cl1	106.53 (2)
C3—C4—N41	119.57 (19)	Cl3^i^—Co1—Cl2	108.21 (3)
C5—C4—N41	118.39 (19)	Cl3—Co1—Cl2	108.21 (3)
C6—C5—C4	118.5 (2)	Cl1—Co1—Cl2	110.81 (4)
C6—C5—H5	120.7		
			
C6—C1—C2—C3	0.8 (3)	C4—C5—C6—C1	0.1 (3)
C11—C1—C2—C3	−177.6 (2)	C2—C1—C6—C5	−0.8 (3)
C1—C2—C3—C4	0.0 (3)	C11—C1—C6—C5	177.7 (2)
C2—C3—C4—C5	−0.7 (3)	C6—C1—C11—O11	−5.0 (3)
C2—C3—C4—N41	178.4 (2)	C2—C1—C11—O11	173.4 (2)
C3—C4—C5—C6	0.7 (3)	C6—C1—C11—C12	175.8 (2)
N41—C4—C5—C6	−178.5 (2)	C2—C1—C11—C12	−5.8 (3)

Symmetry code: (i) −*x*, *y*, *z*.

### Hydrogen-bond geometry (Å, º)

**Table:**

*D*—H···*A*	*D*—H	H···*A*	*D*···*A*	*D*—H···*A*
N41—H41*A*···O11^ii^	0.91 (2)	1.88 (2)	2.781 (3)	174 (3)
N41—H41*B*···Cl2^iii^	0.92 (2)	2.31 (2)	3.211 (2)	168 (3)
N41—H41*C*···Cl3^iv^	0.88 (2)	2.48 (2)	3.309 (3)	157 (3)

Symmetry codes: (ii) −*x*+1/2, *y*−1/2, *z*; (iii) *x*+1/2, *y*, −*z*+3/2; (iv) −*x*+1/2, −*y*+1/2, −*z*+2.

## References

[bb1] Bruker (2004). *APEX2*, *SAINT* and *SADABS*. Bruker AXS Inc., Madison, Wisconsin, USA.

[bb2] Elangovan, A., Thamaraichelvan, A., Ramu, A., Athimoolam, S. & Natarajan, S. (2007). *Acta Cryst.* E**63**, m224–m226.

[bb3] Sheldrick, G. M. (2015). *Acta Cryst.* C**71**, 3–8.

[bb4] Spek, A. L. (2009). *Acta Cryst.* D**65**, 148–155. 10.1107/S090744490804362X PMC2631630 19171970

[bb5] Westrip, S. P. (2010). *J. Appl. Cryst.* **43**, 920–925.

