# Supplementary figures and images for: Crystal structure of bis­(4-acetyl­anilinium) tetra­chlorido­cobaltate(II)
Source: Acta Crystallogr E Crystallogr Commun. 2015 Nov 18;71(Pt 12):m221–2. doi: 10.1107/S2056989015021404 (PMC4719843; doi:10.1107/S2056989015021404)

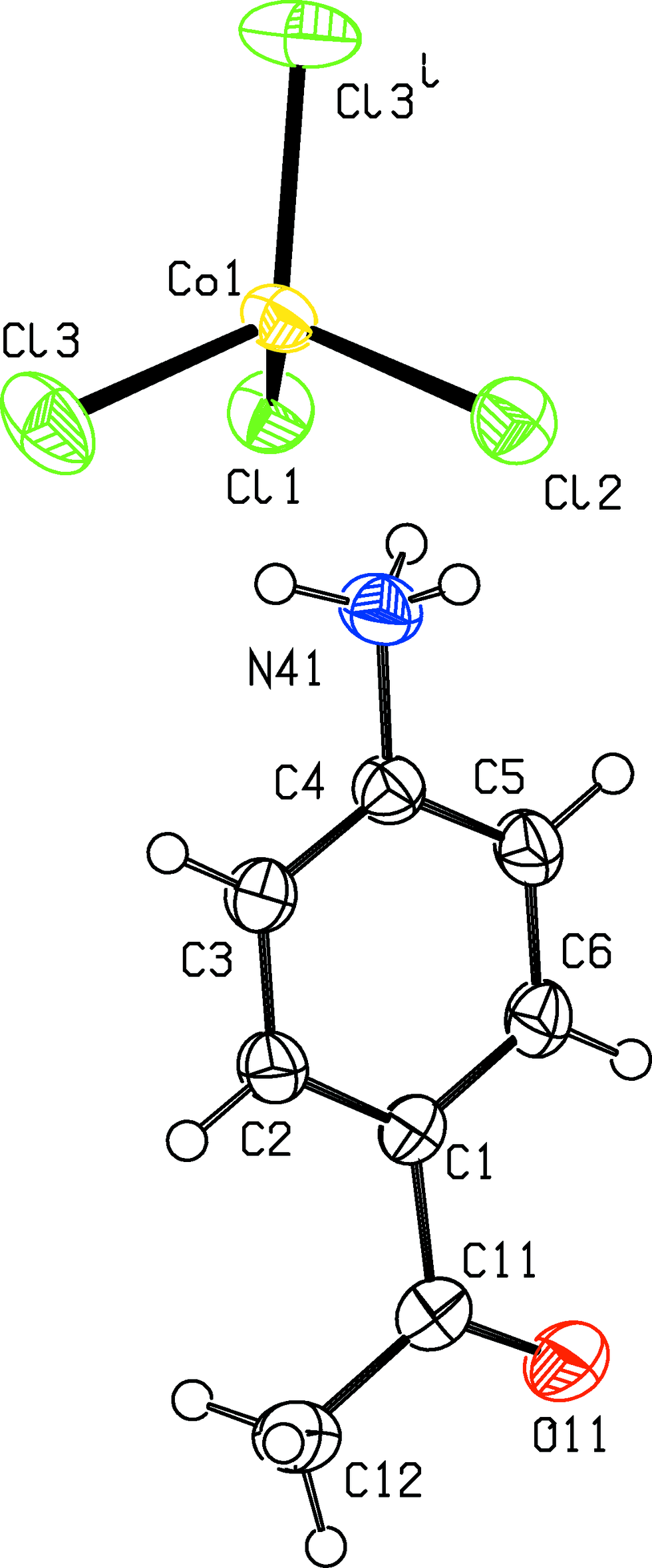

Supplement: Supplementary file 3 [file e-71-0m221-fig1.tif]

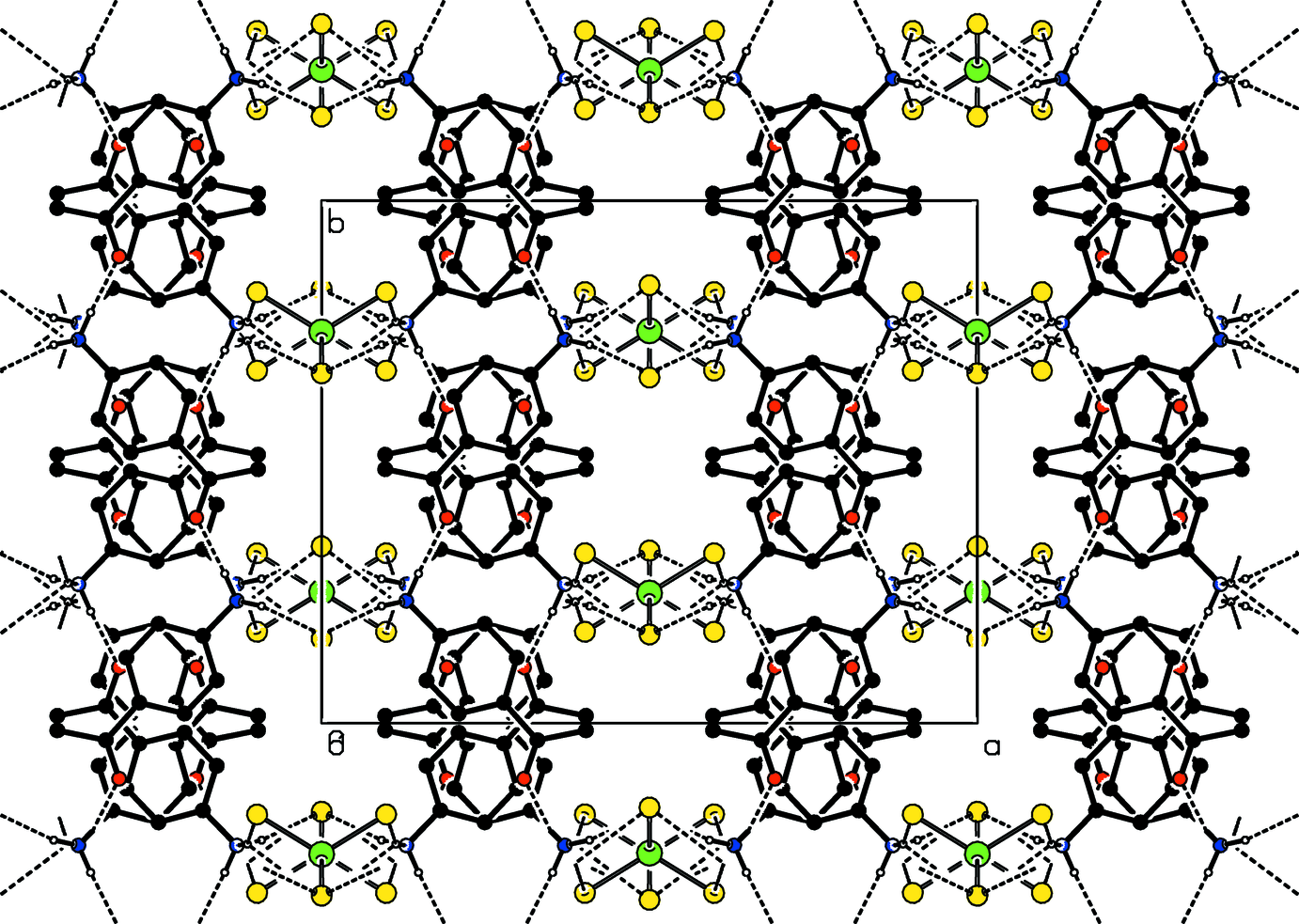

Supplement: Supplementary file 4 [file e-71-0m221-fig2.tif]
